# PEEK Implants: An Innovative Solution for Facial Aesthetic Surgery

**DOI:** 10.1155/2021/5518433

**Published:** 2021-08-05

**Authors:** Rocco Narciso, Emanuela Basile, Davide Johan Bottini, Valerio Cervelli

**Affiliations:** ^1^Plastic Surgery Resident, Graduate School of Plastic Reconstructive and Aesthetic Surgery, “Tor Vergata” University, Rome, Italy; ^2^Department of Plastic and Reconstructive Surgery, Policlinico Casilino Hospital, Rome, Italy; ^3^Reconstructive and Aesthetic Surgery University “Tor Vergata” of Rome, Department of Plastic and Reconstructive Surgery, Policlinico Casilino Hospital, Rome, Italy; ^4^Department of Surgical Sciences, University “Tor Vergata”, Rome, Italy

## Abstract

The authors present a case report showing their experience with the use of PolyEtherEtherKetone (PEEK) implants as an innovative solution for the skeleton and soft tissues' reshaping in facial aesthetic plastic surgery. This technique offers the surgeon a reliable and effective way to answer patients' request of increasing volume and reshaping the malar area. A fifty-year-old patient complaining about hypoplasia of the malar area, after undergoing three operations of silicon implants' placement and replacement, was still unsatisfied about the symmetry and feeling through the skin of the lower lid, the rim of the prostheses. The authors suggested the use of bone-anchored PEEK implants, to increase the volume and reshape the malar area by a skeleton and soft tissue camouflage. The treatment was planned and previewed on the preop 3-dimensional CT scans for the customization of the implants. Although no cases are reported in international literature on the use of this material in facial aesthetic surgery, this technique seems to offer a safe and effective solution for the treatment of patients asking to increase and modify the shape of their malar area. Custom made PEEK implants are already used in craniofacial reconstructive bony surgery with good results, and 3D CT scan planning is widely used in these cases. No complications were reported in the case reported and the outcomes seem to the authors and to the patient being, finally, satisfactory.

## 1. Introduction

The PolyEtherEtherKetone (PEEK) implants are a recent answer to the research of reliable material for facial and skeleton reshaping and camouflage [1]. Different kinds of material can be chosen, and the following are the most popular used today for facial skeleton implantation:

MedPor (high-density porous polyethylene), silicone, titanium, aluminia ceramics, methyl methacrylate, lipofilling, and hydroxyapatite [2].

These implants are utilized to reconstruct several bony and soft-tissue defects, including the frontal and temporal areas; orbital walls; infraorbital margin; zygomatic, paranasal, and nasal regions; and mandible [2].

Craniofacial surgeons often use these materials, performing cranial vault and craniofacial posttraumatic and oncologic bone-defect reconstructions [3].

During surgical planning, the research was oriented to a material that could have a solid structure, a malleable shape, good biocompatibility, and low rate of infection.

The experience with other solution as the methacrylate demonstrates an increase probability of infections and a challenging preoperative implant measures assessment [2]. The PEEK, instead, had less chance of infection and allow an access as the intraoral that is a nonsterile route. The implants are printed with a 3D printer and it is very simple to obtain an implant that perfectly fit the searched volume and shape.

The authors present the case of a patient who underwent three operations of silicon implants' placement and replacement and was still unsatisfied about the symmetry and the feeling of the rim of the prostheses. The patient was treated by the replacement of the old prostheses with PEEK custom made bone anchored implants in the zygomatic area. Customization of the prostheses was planned to preview the shape and the volume to obtain, on the preoperative 3-dimensional CT scans. PEEK has been chosen but rarely described in literature for the reconstruction of the malar regions [4] and is an innovative solution in the field of facial aesthetic surgery.

## 2. Case Presentation

The patient is a fifty-year-old man who received three different surgery procedures for an improvement in projection and reshaping of the malar areas.

In 2014, were positioned bilaterally silicone implant CSM (Combined Malar Shell®) for malar area in the subcutaneous tissues, from an intraoral access, with concomitant malar e submalar lipostructure.

In the 2016, was positioned a new TSM (Terino Malar Shell®) implant near to preexisting one for each side, from an intraoral access.

In 2018, all the protheses were removed and were implanted Hanson SM 12-3® prosthesis for each side.

The authors visited the patient in 2019; he complained about the mobility of the implants and about a subcontinuous nuisance in the infraorbital regions.

The operation performed to achieve the result was the bilateral positioning in the malar area of two custom made PEEK bone-anchored implants. An intraoral and subperiosteal plane approach for both the malar areas places the new prostheses, after removing the old silicone implants.

Based on the preop CT scan, the PEEK PSIs (patient-specific implant) were planned and manufactured previewing the size, shape, and the precise location of the prostheses; even the size and inclination and the type of screw could be planned, in order to achieve a fast and correct placement of the implants.

**Figure 1:**
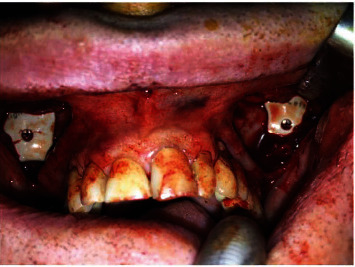
The new PEEK implants fixed into the subperiosteal pocket bilaterally.

Under general anesthesia, the surgical placement was obtained with an incision performed in the upper oral vestibule on preexisting scars and through an intraoral subperiosteal approach. The two preexistent silicone implants were removed, and then, subperiosteal soft tissue detachment is allowed to place the implants on the bone surface and to anchor with screws in the programmed position (Figure 1). The patient was discharged after one night of recovery with an oral antibiotic and analgesic oral therapy.

The follow-up schedule (Table 1) included stitch removal fourteen days after surgery. The postoperative edema showed a great reduction after 2 weeks and a complete resolution after 2 months. A CT scan was performed after 3 months, and photos were taken after 6 and 12 months.

## 3. Discussion and Conclusion

The authors found a satisfactory result, not only aesthetic but also functional with an improvement of the symptoms complained by the patient.

**Figure 2:**
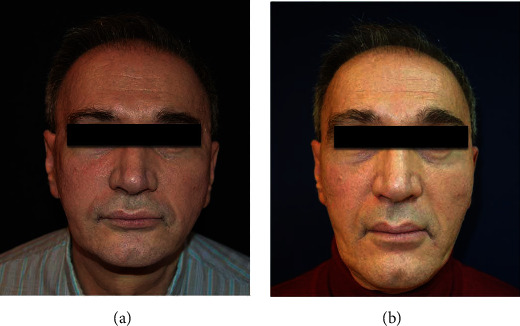
The presurgery picture (a) and the postsurgery picture (b). Published with the patient's consent.

The preoperation pictures and the follow-up pictures after 1 year are shown in the paper (Figure 2).

In the literature, there are several papers that describe the use of the PEEK implants, in orthopedic surgery [5], in maxillo-facial surgery [6, 7], in oral implantology and prosthodontics [8], and in neurosurgery [9]. This material is gaining positive replay for its high biocompatibility and low weight.

Furthermore, the PEEK PSIs can be trimmed with a cutting burr in the operating room if it is needed to be reshaped or if the aesthetic result is not satisfying, despite of the customization process that could not be enough.

PEEK has many advantages compared with other alloplastic implant materials. PEEK has radiographic translucency [10] and does not produce artefacts on radiographic imaging. PEEK is also nonallergenic and nonmagnetic and does not develop exothermic reactions as methyl methacrylate does [3]. Furthermore, PEEK is comparable to cortical bone regarding its elasticity.

The fixation is possible with the traditional screws. The availability of a custom made prothesis allows a significant reduction of the surgery time [4].

The PEEK is widely used in maxillo-facial surgery, but it was never described in aesthetic surgery.

In our case, we found a satisfactory result, not only aesthetic but also functional with an improvement of the symptoms complained by the patient.

## Figures and Tables

**Table 1:** Follow-up schedule.

14 days	Stitch removal
2 months	Edema resolution
3 months	CT scan control
6 months	Photo control
12 months	Photo control
